# Heterogeneity Detection Method for Transmission Multispectral Imaging Based on Contour and Spectral Features

**DOI:** 10.3390/s19245369

**Published:** 2019-12-05

**Authors:** Yanjun Wang, Gang Li, Wenjuan Yan, Guoquan He, Ling Lin

**Affiliations:** 1 State Key Laboratory of Precision Measuring Technology and Instruments, Tianjin University, Tianjin 300072, China; 2117202062@tju.edu.cn (Y.W.); ligang59@tju.edu.cn (G.L.); 2 Tianjin Key Laboratory of Biomedical Detecting Techniques and Instruments, Tianjin University, Tianjin 300072, China; 3 School of Electronic Information Engineering, Yangtze Normal University, Chongqing 408100, China; 20000032@yznu.cn (W.Y.); 19000111@yznu.cn (G.H.)

**Keywords:** heterogeneity detection, spectral feature, transmission multispectral imaging (TMI), image exponential downsampling

## Abstract

Transmission multispectral imaging (TMI) has potential value for medical applications, such as early screening for breast cancer. However, because biological tissue has strong scattering and absorption characteristics, the heterogeneity detection capability of TMI is poor. Many techniques, such as frame accumulation and shape function signal modulation/demodulation techniques, can improve detection accuracy. In this work, we develop a heterogeneity detection method by combining the contour features and spectral features of TMI. Firstly, the acquisition experiment of the phantom multispectral images was designed. Secondly, the signal-to-noise ratio (SNR) and grayscale level were improved by combining frame accumulation with shape function signal modulation and demodulation techniques. Then, an image exponential downsampling pyramid and Laplace operator were used to roughly extract and fuse the contours of all heterogeneities in images produced by a variety of wavelengths. Finally, we used the hypothesis of invariant parameters to do heterogeneity classification. Experimental results show that these invariant parameters can effectively distinguish heterogeneities with various thicknesses. Moreover, this method may provide a reference for heterogeneity detection in TMI.

## 1. Introduction

Transmission multispectral imaging (TMI) can be used for early breast cancer detection because heterogeneous materials may be detected by using multiband information [1,2,3,4]. Unfortunately, biological tissue has strong absorption and scattering characteristics, which results in a low signal-to-noise ratio (SNR) and blurry images. Existing research has focused on improving the SNR to produce higher quality images that enable tumor detection. Dhokkar [5,6] proposed a frame-accumulation method that improves the SNR of the image. Then, Li [7,8] and Tang [9] proposed a method of combining frame-accumulation technology with shape function signal technology in the preprocessing stage. This method improves the SNR of the image. Thomas [10] proposed a method of using a low-rank structure of biological tissue data to capture intra-wave frequency modulation signals. To improve light-emitting diode (LED)-based multispectral image acquisition, Li [11] proposed and proved the multiwavelength “synergy effect”, which can improve the quality of each waveband obtained by frequency-division modulation. TMI may become an effective detection method when supported by the frame-accumulation method, shape function signal modulation/demodulation technology and the multiwavelength “synergistic effect”.

In medical image processing, researchers pay more attention to image segmentation. Adhikari [12] proposed a magnetic resonance imaging (MRI) segmentation method that combined several conditioning effects using a conditional variable for each pixel and aggregating spatial information into membership functions that were then used as restriction conditions of a fuzzy c-means algorithm. By leveraging the specificity of phase-contrast signals in computed tomography images, Burn [13] proposed a segmentation method based on a viscous watershed transform, which segments tumors into phase contrast mammography images created via a high-resolution X-ray analyzer. Although the image definition of TMI is poor compared to existing detection methods, it has the advantage of multiwavelength information. In the early stages of a tumor, analyzing the characteristics of a suspicious area is often more valuable than contour extraction. If a suspicious area is detected, professional diagnosis and treatment can be carried out. Zhang [14] has focused on heterogeneity detection to classify two different substances by using Faster Region-based Convolutional Network (Faster-RCNN) and Single Shot MultiBox Detector (SSD) methods to train a set of 15,000 multispectral transmission image samples.

Medical image processing often has to rely on small data sets. We develop a method that doesn’t require a large data set. First, we use frame accumulation and shape function signal modulation and demodulation technology to improve image quality. Then, a heterogeneity detection method is proposed that includes heterogeneity contour extraction and classification. In the contour-extraction step, we use an image exponential downsampling pyramid and Laplace operator to roughly extract and fuse the contour of any heterogeneities in different wavelength images. Then, we can use the inferences of an invariant ratio of absorption and the scattering characteristics of different wavelengths to do heterogeneity classification. Experimental results show that it is easy to do heterogeneity classification by using this method. Moreover, this method reduces the difficulty of heterogeneity classification.

## 2. Theories and Methods

### 2.1. Frame Accumulation and Shape Function Signal Modulation and Demodulation Technologies

Resolution is the core of image accuracy and heterogeneity detection sensitivity [15]. The higher the grayscale resolution of an image, the richer the image information, and the better the classification and analysis of the material. In the image acquisition process, SNR and grayscale are mainly affected by random noise. The impact of random noise can be reduced by using the frame-accumulation method. If the sampling point is *N*, the mean value of the signal can be defined by Equation (1) [5,6].

(1) $$\bar{x}=\frac{1}{N}\sum_{i\;=\;1}^{N}\left(x\;+\;\Delta x_{i}\right)\;+n=x+\frac{1}{N}\sum_{i\;=\;1}^{N}\Delta x_{i}+n$$

where *n* is fixed pattern noise, *x* is the true value, and $\Delta x_{i}$ is the random error between sampling value and true value. With the increase of sampling points, the intensity of the random noise will decrease gradually.

Multiwavelength function signal modulation/demodulation is the process of moving the spectrum from its baseband signal to develop a system of multiple wavelengths and a channel passband of different frequencies. The band signal is restored by the baseband signal via a Discrete Fourier Transform (DFT). The use of these methods provides high-quality images for heterogeneity detection.

### 2.2. Heterogeneity Detection for TMI

#### 2.2.1. Rough Contour Extraction Algorithm of Heterogeneity

For TMI, the contour of heterogeneity is a transitional range. A downsampling method can narrow this boundary, making the image contour easier to extract. Thus, we propose a kind of rough contour-extraction algorithm. The algorithm mainly includes the image exponential downsampling method and Laplacian filtering.

**Figure 1:**
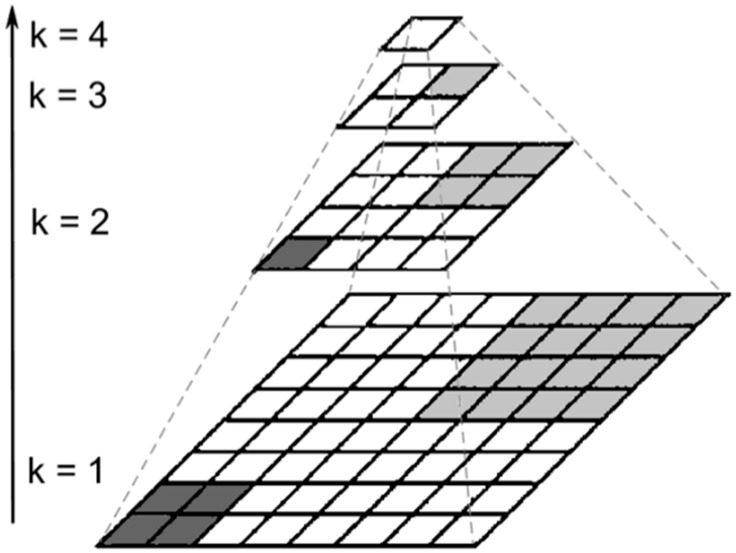
A 2-based exponential downsampling diagram. Each square represents a pixel that is sampled every time and accumulated every 4 adjacent points.

The idea of the image exponential downsampling pyramid comes from the Gauss image pyramid [16,17] and spatial pixel-binning [18]. The purpose of exponential downsampling is to sacrifice the spatial resolution of the image to improve the contrast of boundary information. We consider 2-based image exponential downsampling pyramid as an example. Here are the steps:

(1) Let *k* represent the number of layers that will be downsampled and define the original image as the first downsampling layer, denoted by *k* = 1.
(2) Let the accumulation of 4 (if it is 3-based, the number will be 9) adjacent pixels correspond to a single pixel in the next layer. Any pixels that are accumulated in this layer will not participate in this layer accumulation again. If a row or column is left at the end of the layer, it is deleted.
(3) Step (2) remains in a loop until the number of rows and columns equals 1, as shown in Figure 1.

A Laplacian operator can be used as a rotation-invariant isotropic boundary operator. After Laplacian filtering, the location of the boundary is found to be near the origin, and the outline of the suspicious regions can be approximately obtained by using a zero point as the boundary point. By using Laplacian filtering for all downsampled sequences, we can obtain approximate regions where heterogeneity may exist in any band at higher grayscale resolution. Thus, a second-order differential linear operator, the Laplacian operator, is used to enhance the edges of the layers. For two-dimensional images, a Laplacian operator is defined as

(2) $$\nabla^{2}f=\left(\nabla^{2}f\right)/\left(\nabla x^{2}\right)\;+\;\left(\nabla^{2}f\right)/\left(\nabla y^{2}\right)$$

there are two common adjacency matrices used for Laplacian operators, one is the 4-vertex adjacency matrix and the other is the 8-vertex adjacency matrix. The 8-vertex adjacency matrix is more stable and more commonly used than the 4-vertex adjacency matrix. Equation (3) is the 8-vertex adjacency matrix for Laplace templates:

(3) $$\nabla^{2}f=\sum_{i=-1}^{1}f\left(x+i,y+j\right)-9f\left(x,y\right)$$

#### 2.2.2. Beer–Lambert’s Law and Spectral Feature Analysis

Beer–Lambert’s law [19] is the basic law of spectrophotometry. It revealed that when a beam of light passes through a material, some of the components of the beam will be absorbed and the absorption spectrum of the substance can be obtained from the spectrometer. Almost all substances have their unique absorption spectra. The absorption spectra are characterized by Equation (4):

(4) A=lg(1/T)=Kbc

where *A* is the absorption, *K* is the molar absorption coefficient, *c* is the concentration of the light absorbing substance in mol/L, *T* is the transmittance, which is the ratio of transmitted light intensity to incident light intensity, and *b* is the thickness of the absorption layer in cm. Its physical significance can be described as follows: when a beam of parallel monochromatic light vertically passes through a uniform non-scattering light absorber, its absorbance is proportional to the concentration of the light absorber and the thickness of the absorption layer, and inversely proportional to the transmittance.

Different substances have different spectral properties for different wavelengths [20]. Biological tissue has strong absorption and scattering characteristics [4,21], so the attenuation of light flux after the light source passes through a heterogeneous area is mostly determined by absorption and scattering characteristics. Hence, we made a hypothesis: the attenuation of light flux varies with the thickness of the heterostructure, but the ratio of attenuation value of luminous flux between different wavelengths of the same substance may be constant or fluctuate over a small range. The absorption coefficient of fat is much lower than that of a tumor and other breast-tissue components [22,23,24,25]. Thus, we consider fat as background and ignore its gray-level changes. Then, the background is subtracted from the original image to obtain the light flux attenuation value. Finally, we use the attenuation of light flux to classify different heterogeneities. If we use three wavelengths, the distinguishing parameters can be defined as:

(5) $$Pm_{x}=\frac{Wave_{1}}{Wave_{2}}\;\;Pm_{y}=\frac{Wave_{2}}{Wave_{3}}$$

where the $Wave_{1}$ is the flux attenuation value of the first wavelength light source passing through heterogeneity. The definition of $Wave_{2}$ and $Wave_{3}$ are the same as $Wave_{1}$. The $Pm_{x}$ and $Pm_{y}$ are the two parameters we use to characterization a heterogeneity.

## 3. Experiment

**Figure 2:**
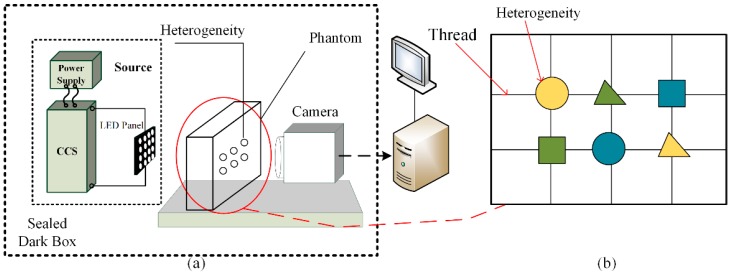
Experimental system structure. (**a**) Experimental system structure diagram. (**b**) Phantom structure picture. The six heterogeneous samples are fixed by the stent in the fat emulsion.

A schematic diagram of an experimental system, for the theoretical method described in Section 2, is shown in Figure 2. The device consists of the following components: three wavelength LEDs (blue wavelength: 490–495 nm, green wavelength: 520–525 nm, red wavelength: 620–625 nm), three multifunctional signal generators (MFSG), three constant current sources (CCS), an industrial camera (model: JHSM500f, maximum resolution: 2592 × 1944 pixels, sensitization device: Complementary Metal Oxide Semiconductor (CMOS)), a computer (connected to an industrial camera), shading cloth (to isolate the environment light), and phantom material. The modulator uses a step-down constant current source driver board (Model: PT4115). The phantom material is composed of a high transmittance polymethyl methacrylate (PMMA) cuboid container (size: 150 mm × 110 mm × 500 mm), a 3% concentration of fat emulsion injection (C14~24, original concentration: 30%), a stent used to fix the heterogeneous samples, and an array of heterogeneous samples. The heterogeneous samples include three sets of raw pork pieces (the muscle part), pumpkin pieces and potato pieces with different thicknesses. The thickness of all thin heterogeneous materials is 3–5 mm, and the thickness of all thick heterogeneous materials is 7–9 mm.

(A) Data acquisition
  (1) Set up the equipment according to Figure 2. The distance between the industrial camera and the phantom is set to 400 mm, and the distance between the LED array and the phantom is set to 300 mm. The frequency of the modulated three-wavelength source is set to 0.5 Hz, 2 Hz and 8 Hz. The sampling frequency of the camera is set to 32 Hz. The pre-constructed heterogeneous array will be placed 20 mm away from the wall of the container on the side of the industrial camera.
  (2) The pre-constructed heterogeneous sequence is fixed into the fat emulsion. Ten frames of images were taken to obtain the true contour. Inject the fat emulsion into the container, turn on the light source, and cover the shading cloth. Capture 2400 frames of modulated images.
  (3) Turn off the modulation signal and remove the heterogeneous sample. Use another set of samples to repeat step (2) twice. Finally, turn off the modulation signal and shoot a 2400 frame multiwavelength image of the pure fat emulsion.
(B) Data processing
  (1) The three-wavelength transmission image is demodulated using a frequency-domain fast demodulation algorithm [26].
  (2) For each group of data, compute the 2-based exponential downsampling pyramid of the demodulated image. Apply the Laplacian operator to filter the exponential downsampling sequence. All pixels are binarized with a threshold of 0.
  (3) Extract the first and second images without salt-and-pepper noise from the exponential downsampling pyramid. Hence, according to the downsampling path, use the cubic polynomial interpolation method to upsample these two images to their original image size, and calculate the union. Do a union operation to the resulting three-wavelength image and merge the heterogeneous rough outline.
  (4) Corrode the nonheterogeneous area and define the average value of the rest of the nonheterogeneous area as the approximate gray value of the background. The attenuation value of light flux is obtained by subtracting the approximate background from the original image. Finally, the invariable characteristics of $Pm_{x}$ and $Pm_{y}$ are calculated by Equation (4).

## 4. Results and Discussion

### 4.1. Evaluation of Image Acquisition Method

**Figure 3:**
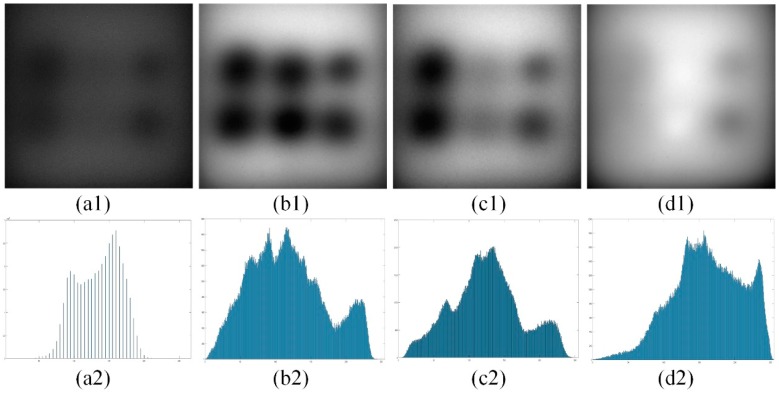
One set of image data. All images were stretched and displayed at 8 bit. (**a1**) A random single frame image, (**b1**) blue wavelength image after demodulation, (**c1**) green wavelength image after demodulation, (**d1**) red wavelength image after demodulation, (**a2**) gray histogram of a1, (**b2**) gray histogram of b1, (**c2**) gray histogram of c1, (**d2**) gray histogram of d1.

The SNR and grayscale levels of the demodulated images obtained by using frame accumulation and shape function signal modulation and demodulation technology are significantly improved compared with the single-frame image. Three sets of image data are obtained through data processing step (1), where Figure 3a1 is a random single frame image, and Figure 3b1–d1 is a single set of demodulated images. The specific location and thickness data of heterogeneous materials are pork (thin), pumpkin (thin), potato (thin), pork (thick), pumpkin (thick), and potato (thick). As can be seen from Figure 3, the grayscale level of the demodulated image is significantly improved compared to a single-frame image. In addition, we normalize the image data and use SNR based on the human visual system (HSNR) [27], a point-acuity-based entropy function method (EFM) [28], and image entropy three no-reference image quality assessment (IQA) criteria to do the IQA in Table 1. The mean values of the three-wavelength images under the three evaluation criteria are 1.091, 5.391, and 7.603. Compared with one single-frame image, the average scores of the three-wavelength images are increased by 443.8%, 73.2% and 66.0%, respectively. This improvement of image quality makes heterogeneity detection possible in TMI.

### 4.2. Evaluation of Image Detection Method

**Figure 4:**
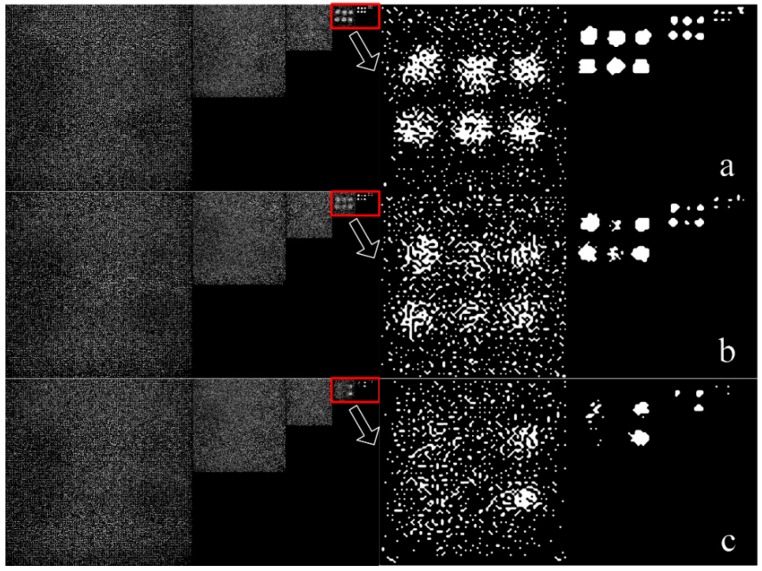
One set of 2-based exponential downsampling pyramid after Laplacian filtering and binarization. From left to right are graphs where *k* = 1,2,3…8. (**a**) Blue wavelength, (**b**) green wavelength, (**c**) red wavelength.

(A) On the basis of image acquisition method, the rough contour segmentation method can detect all heterogenous regions. Figure 4 is the image data obtained by data processing step (2). Image sequences with small k values are filled with salt-and-pepper noise. As the spatial resolution decreases, the blurred outline becomes clearer in the red box.

**Figure 5:**
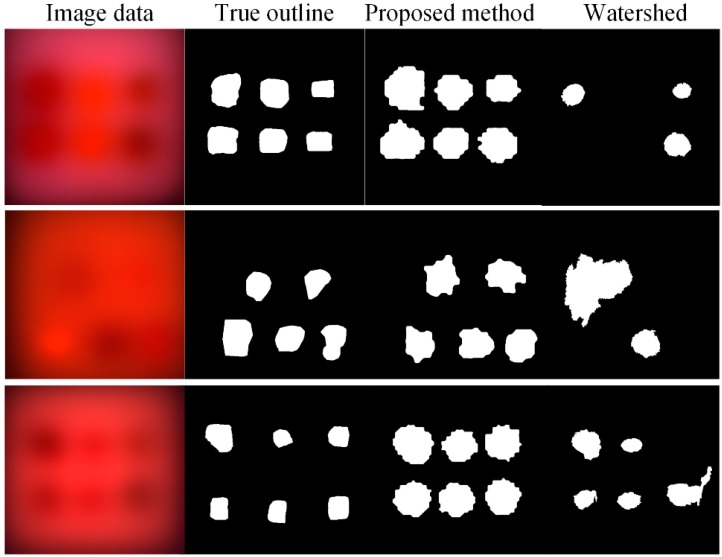
Three sets of demodulated images, true outlines, and outlines predicted by the proposed method and watershed algorithm.

**Figure 6:**
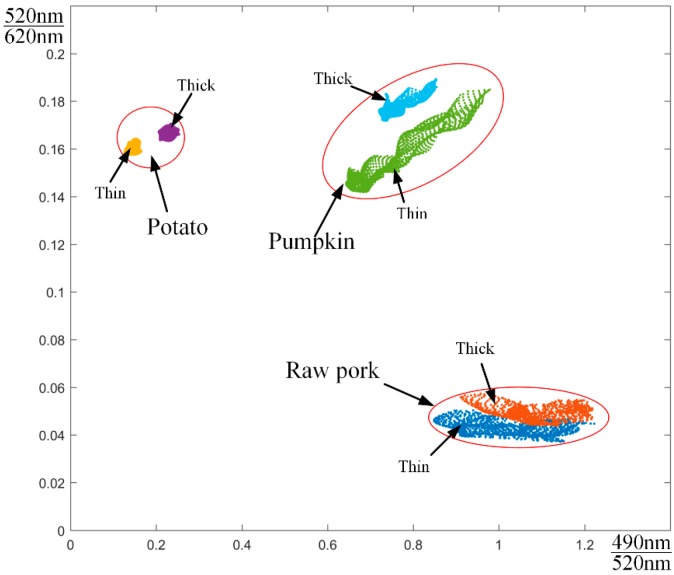
Scatter plot of relative ratios of attenuations at different wavelengths for different heterogeneities.

Figure 5 shows the outline of heterogeneity obtained by data processing step (3) and watershed algorithm [29]. The ‘image data’ is the image obtained by data processing step (1). As can be seen from Figure 4, the watershed algorithm misses some hard-to-detect contours in contour prediction. The rough contour segmentation method obtains a rough outline of all heterogeneities. The image exponential downsampling pyramid decomposes the fuzzy image into different scales. As the spatial resolution shrinks, the scattered image boundaries converge, making it easier for rough contour extraction. What is more, multiwavelength contour information is merged to ensure that all contour information is used. Compared with the watershed algorithm, the proposed rough contour-extraction algorithm effectively uses multispectral information and image spatial information.

(B) Based on the unique spectral characteristics of heterogeneity, this classification method can work effectively. The attenuation value of light flux is calculated through data processing step (4). In this example, we use the attenuation at blue wavelength to represent $Wave_{1}$, the attenuation at green wavelength for $Wave_{2}$, and the attenuation at red wavelength as $Wave_{3}$. The ratio scatter plot is drawn in Figure 6, where the x-coordinate is $Pm_{x}$ and the y-coordinate is $Pm_{y}$. The mean value of the attenuation ratio of the region is listed in Table 2.

As shown in Figure 6, we immediately find that heterogeneous regions are clearly divided into three categories. The lower right corner of the figure shows the samples of raw pork (with different thicknesses). The left side of the figure shows potato samples, and the upper right corner is pumpkin samples. Heterogeneous samples with the same characteristics are grouped together. Potato and pumpkin cannot be distinguished by combining the green wavelength and red wavelengths, but they can be separated by adding a blue wavelength. Table 2 shows the mean values of the ratios of the three heterostructures at different wavelengths. These values represent the unique characteristics of the three heterogeneous materials in this experimental environment.

However, the ratio in Figure 6 is a point cloud, not a point. To be exact, the wavelength of the LED in this experiment is technically a wave band (blue wavelength: 490–495 nm, green wavelength: 520–525 nm, red wavelength: 620–625 nm), and the exact ratio is (490–495 nm)/(520–525 nm). Additionally, the actual experiment could not ensure that the temperature, heterogeneous purity and fat-emulsion concentration are absolutely unchanged. Without considering these errors, this result demonstrates that different heterogeneous samples have different absorption and scattering effects at different wavelengths. Moreover, in the case where the total amount of light intensity is constant, the ratio of the relative luminous flux between different wavelengths of the same substance fluctuate over a small range. After obtaining the corresponding parameters, any classification methods can be used to assist in the heterogeneity classification, such as neural network, support vector machine (SVM), and clustering. This method reduces the difficulty of heterogeneity classification.

## 5. Conclusions

In this work, we focused on mitigating the difficulties brought about when trying to detect heterogeneous materials from TMI. We used frame accumulation and shape function signal modulation and demodulation technology to improve image quality. Then, a kind of heterogeneity detection method based on contour features and spectral features was proposed. In the fuzzy image contour-extraction step, we provided a kind of composite contour-extraction method to extract contours from strong scattering images. Then, we used the ratio of the heterogeneous luminous flux attenuation value of different wavelengths to do heterogeneity classification. This classification method is not affected by heterogeneous thickness. Moreover, this combination method may provide a reference for TMI in medical applications.

## Figures and Tables

**Table 1:** Image quality evaluation results of Figure 3a1–d1.

Image Data	HSNR	EFM	Image Entropy
**a1**	0.20106	3.11246	4.57925
**b1**	1.01079	4.98533	7.67579
**c1**	1.09825	5.43018	7.58585
**d1**	1.16403	5.75690	7.54877

**Table 2:** The mean of the ratio of the luminous flux attenuation of each wavelength.

Heterogeneity	490 nm/520 nm	520 nm/620 nm
**Raw pork**	1.0637	0.0429
**Potato**	0.1954	0.1733
**Pumpkin**	0.8018	0.1610
